# Dysregulation of Receptor for Advanced Glycation End Products (RAGE) Expression as a Biomarker of Keratoconus

**DOI:** 10.1155/2022/1543742

**Published:** 2022-01-15

**Authors:** Valentin Navel, Jean Malecaze, Corinne Belville, Héléna Choltus, Fanny Henrioux, Frédéric Dutheil, François Malecaze, Frédéric Chiambaretta, Loïc Blanchon, Vincent Sapin

**Affiliations:** ^1^University Hospital of Clermont-Ferrand, CHU Clermont-Ferrand, Ophthalmology, F-63000 Clermont-Ferrand, France; ^2^Translational Approach to Epithelial Injury and Repair Team, University of Clermont Auvergne, CNRS UMR 6293, INSERM U1103, Genetic Reproduction and Development Laboratory (GReD), F-63000 Clermont-Ferrand, France; ^3^University of Clermont Auvergne, CNRS, LaPSCo, Physiological and Psychosocial Stress, CHU Clermont-Ferrand, University Hospital of Clermont-Ferrand, Preventive and Occupational Medicine, Witty Fit, F-63000 Clermont-Ferrand, France; ^4^Ophthalmology Department, Pierre-Paul Riquet Hospital, Toulouse University Hospital, Toulouse, France; ^5^Department of Biochemistry and Molecular Genetic, CHU Clermont-Ferrand, F-63000 Clermont-Ferrand, France

## Abstract

**Background:**

Because of the implications of Receptor for Advanced Glycation End Products (RAGE) in keratoconus (KC), we describe a differential expression of RAGE transcripts and proteins in corneal tissues and tears of KC and healthy patients.

**Methods:**

Using a case-controlled study, corneal epitheliums and tears of KC and healthy subjects were obtained during corneal collagen cross-linking and photorefractive keratectomy (PKR) and during usual consultations. Quantitative reverse transcription (RT-qPCR) and Western-Blot were performed to analyze RAGE transcripts and proteins' expression in corneal tissues and tears.

**Results:**

One hundred and six patients were included in this study. The characteristics of the patients were as follows: 56 KC (25 corneal epithelium and 31 tears) and 50 control subjects (25 corneal epithelium and 25 tears). Transcripts of RAGE, HMGB1, and S100 family ligands were quantified by RT-qPCR, identifying a significantly higher expression of RAGE and HMGB1 in the healthy group than in the KC group (*p* = 0.03 and 0.04, respectively). Western Blot showed a significantly higher fl-RAGE expression in KC corneal epithelium than control (*p* < 0.001) and lower s-RAGE expression in KC tears than control (*p* = 0.04).

**Conclusions:**

Linked with the inflammatory process occurring in KC pathophysiology, we propose for the first time that the RAGE expression (total and truncated forms of receptor and ligands) in KC corneal tissues and tear samples provides viable biomarkers.

## 1. Introduction

Keratoconus (KC) is a progressive vision-threatening corneal disease classically beginning in the second decade of life, causing deformation of the structure of the cornea [1–4] Because the regular corneal curvature has a predominant role in refraction and quality of vision [5], this disease causes a visual impairment despite therapeutic strategies involving the use of rigid contact lenses [6], corneal collagen cross-linking [7, 8], refractive surgeries [9], or advances in corneal grafting [10, 11]. The prevalence of KC is approximately 5.3/2000 in the general population [12], with certain ethnic groups, such as Asians being more at risk [13]. Well-known environmental factor such as eye rubbing [14, 15] or atopy [16, 17] also increases the frequency. There is some evidence for genetic transmission [18–20]. However, the physiopathology of this disease is still poorly understood, and many questions persist in relation to triggering and progression of ectasia. To our knowledge, there are no biomarkers clearly described in the ocular surface (e.g., corneal tissues or tears) of KC. If KC was initially defined as a noninflammatory corneal disease, several studies during the past decade have a described inflammatory process in corneal tissues and tears. In fact, proinflammatory cytokines (IL-1, IL-6, TNF-*α*, and TGF-*β*), oxidative stress, or degradation of collagens by metalloproteases (MMP-1, MMP-3, and MMP-9) has been implicated in keratoconus pathophysiology [3, 21–25].

**Figure 1:**
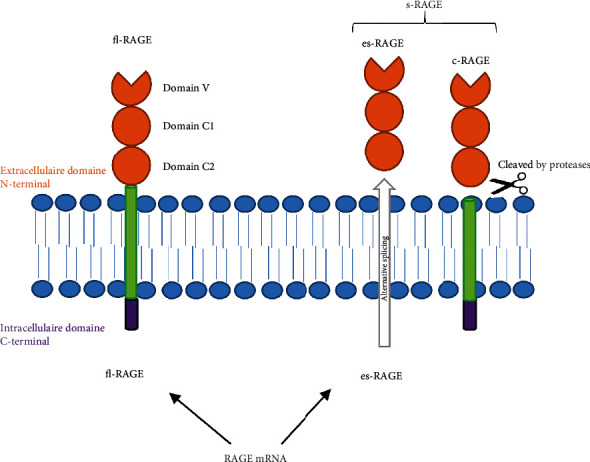
RAGE isoforms: transmembrane receptor and soluble forms.

Receptor for Advanced Glycation End Products (RAGE) is a multiligand transmembrane receptor (member of the immunoglobulin superfamily) implicated in inflammation and cell migration processes [26–28]. The pre-mRNA of RAGE can be subjected to alternative splicing causing truncated RAGE isoforms from the same gene: full-length RAGE (fl-RAGE) and soluble forms RAGE (s-RAGE) including endogenous secretory RAGE (es-RAGE) and cleaved RAGE (c-RAGE), with molecular weights of 35-55 kD varying between isoforms, cellular types, and tissues. fl-RAGE is a transmembrane receptor (able to transduct the signal) whereas s-RAGE corresponds to soluble truncated isoforms of fl-RAGE, comprising an extracellular domain, and plays a role as a decoy to prevent ligands from interacting with fl-RAGE receptors [29–31] (Figure 1). Expression of RAGE, described in several human tissues, is upregulated in response to its natural and historical ligand, i.e., advanced glycation end products (AGEs), but also HMGB1 and S100 proteins that are well-known in aseptic inflammation and oxidative stress of diabetes patients, atherosclerosis, pulmonary and auto-immune diseases, cancer, and chronic neurodegenerative like Alzheimer's disease [30–34]. In ophthalmology, we recently demonstrated the implications of RAGE in the first steps of corneal epithelial wound healing [35].

In addition, previous studies described the role of RAGE in primary open-angle glaucoma [36], diabetic retinopathy [37–39], age-related macular degeneration [40], endothelial Fuchs's dystrophy [41], cataract, and posterior capsule opacification [42] or pterygium [43]. However, to our knowledge, there is no description in KC. Therefore, the purpose of this study was to highlight the role of the RAGE pathway in the cornea and tears of KC compared to healthy patients. A further aim was to describe a putative candidate viable biomarker in the ocular surface of KC.

## 2. Material and Methods

### 2.1. Subjects

One hundred and six subjects, over 18 years of age, were enrolled in the study. Fifty healthy subjects and 56 patients with clinically and topographic KC were considered for the study. The exclusion criteria were ocular surgery performed during the past 3 months, ocular allergy or any ocular disease, dry eye syndrome, any systemic disease, and local or systemic medication (anti-inflammatory) that could interfere with the interpretation of the results. The diagnosis of KC was performed by clinical examination (objective and subjective refraction, slip lamp with fluorescein testing, and tonometry) and topographic evaluation using a Pentacam (Oculus Optikgeräte GmbH, Wetzlar, Germany). The research received approval from the University Hospital Ethics Committees and was conducted in accordance with the recommendations of the Declaration of Helsinki on Biomedical Research Involving Human Subjects. Prior to any data collection, all patients were orally informed about the nature of the study and the working hypothesis and therapeutic education concerning keratoconus. Signed informed consent was obtained from all patients prior to collection of tears or epithelium samples.

### 2.2. Corneal and Tear Sampling

Corneal epitheliums of KC were obtained during surgical intervention of collagen cross-linking with UVA irradiation (A-CXL) and corneal de-epithelialization (Epi-Off technique). After topical anaesthesia using one drop of Oxybuprocaine (Oxybuprocaine THEA 1.6 mg/0.4 ml, single-dose eye drop), we used a disposable scarifier to deepithelialize 6.5 to 8.5 mm of cornea. Epithelial samples were stored at -80°C in 1.5 ml Eppendorf tubes. The same protocol was used for healthy subjects undergoing PRK surgery.

Tear samples were collected without topical anaesthesia, using calibrated 20 *μ*l glass micropipettes (BLAUBRAND intraMark, Wertheim, Germany), from the inferior temporal tear meniscus, taking care to minimize the irritation of the ocular surface. The samples (from 5 to 8 *μ*l per eye) were placed in 0.2 ml Eppendorf tubes using a pipetting aid for capillaries (HIRSCHMANN Laborgeräte GmbH & Co., Eberstadt, Germany) and stored at −80°C.

### 2.3. RNA Extraction and Reverse Transcriptase Quantitative PCR Assays

**Figure 2:**
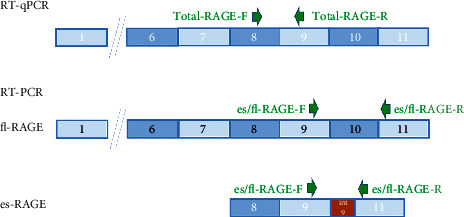
Amplification of total RAGE and its two principal isoforms (fl-RAGE and es-RAGE) by RT-qPCR. Numbers represent exons; the green arrows involve the forward (F) and reverse (R) transcriptions.

Total RNA was extracted from corneal epithelium using RNeasy® Mini Kit (Qiagen, 74106). The RNA concentration was determined by spectrophotometry at 260 nm with the Denovix DS-11 FX NanoDrop. The cDNA was synthetized from 1 *μ*g of RNA using oligo-(dT) primers (Promega, C1101), 10 mM dNTP (Invitrogen, 10297-018), SuperScript IV Reverse Transcriptase (Invitrogen, 18090050), and rRNasin® RNase Inhibitor (Promega, N2515). The reaction mixture (20 *μ*l) was incubated at 65°C for 5 min, 50°C for 10 min, 80°C for 10 min, and, after addition of RNase H (Promega, M4281), 37°C for 20 min. The primer sequences used for classic or quantitative RT-PCR are indicated in Table 1. Recombinant Taq Polymerase (Invitrogen, 10342-020) and 5 mM dNTP (Invitrogen, 10297-018) were used. The reaction mixture (50 *μ*l) was incubated for 10 min at 95°C, followed by 35 amplification cycles—comprising 45 sec at 94°C, 45 sec at the annealing temperature specific to each primer pair, indicated in Table 1, and 45 sec at 72°C and terminated by 10 min at 72°C. A negative control for amplicon contamination was set up using a complete PCR mix without cDNA. Quantitative PCR reactions were performed using LightCycler® 480 SYBR Green I Master (Roche, 04887352001). We applied the following program on the LightCycler® 480 Instrument II (Roche): 10 min at 95°C, followed by 45 amplification cycles comprising 10 sec at 95°C, 10 sec at the annealing temperature specific to each primer pair, indicated in Table 1, and 15 sec at 72°C. PCR reactions were assessed using Taq DNA polymerase (Thermo Fisher Scientific, EP0405). We applied the following procedure: 56°C for hybridization, 5 min at 95° followed by 35 amplification cycles—comprising 45 s at 94°C, 45 sec at the annealing temperature specific to each primer pair, indicated in Table 1, and 45 sec at 72°C and terminated by 10 min at 72°C. Results were analyzed on a 2% agarose gel. (Figure 2) Quantification of two housekeeping genes, *RPLP0* and *RPS17*, and transcripts was performed for all samples as an internal control of the amount and quality of cDNA. These two genes were giving the same results when used separately to standardize. Standard curves were used to quantify the number of amplified transcripts. The results are given as the ratio between the amount of each *transcript of interest* and the geometric mean of these two housekeeping genes (*RPLP0 and RPS17)* as recommended by the MIQE guidelines. All experiments were performed in triplicate.

### 2.4. Protein Extraction and Quantification

The corneal epithelium was, respectively, lysed with either 500 *μ*l or 250 *μ*l RIPA buffer (20 mM Tris (pH 7.5), 150 mM NaCl, 1% Nonidet P-40, 0.5% Sodium Deoxycholate, 1 mM EDTA, and 0.1% SDS), supplemented with 10% Protease inhibitor cocktail (Roche, 04693159001). For the tissues, a preliminary disruption step was performed using ceramic beads (Precellys, KT03961-1-009.2) and a tissue lyser (Qiagen) (three lysing steps during 25 sec with a 30 Hz oscillation frequency, separated by two break steps during 30 sec). Samples were then vortex-mixed for 5 sec and kept on ice for 10 min, one in three occasions. After centrifugation (5 min, 8000 rpm), supernatants were collected. The Pierce™ BCA Protein Assay Kit (Thermo Fisher Scientific, 23225) was used to measure the protein concentration according to the manufacturer's guidelines.

### 2.5. Western Blot Assays

Forty micrograms of proteins were separated a 4–15% Mini-PROTEAN™ TGX Stain-Free™ Protein gel (Bio-Rad) and transferred to nitrocellulose membranes (Bio-Rad, 1704271) using the Trans-Blot® Turbo™ Transfer System (Bio-Rad). After saturation with 5% skimmed-milk in 1X Tris-Buffered Saline (TBS) buffer (blocking buffer) for 2H, membranes were incubated with primary antibody, anti-RAGE polyclonal goat IgG (1 : 5000 dilution in blocking buffer supplemented with 0.1% Tween-20) (Biotechne, R&D Systems France, AF1179), overnight at 4°C. Membranes were washed three times for 10 min with 1X TBS-0.1% Tween-20 (TBST) and incubated with a 1 : 5000 dilution of peroxidase-conjugated polyclonal anti-IgG goat (Abliance, BI2403) for 2 h. Blots were washed with TBST on three occasions for 10 min, rinsed with 1X TBS, and developed with the Clarity Max™ Western ECL Blotting Substrates (Bio-Rad, 1705062) according to the manufacturer's protocols. The All Blue Standard (Bio-Rad, 161-0373) was used as a protein ladder. The relative intensities of protein bands were analyzed using Image Lab™ software (BIO-RAD), and the results were presented as a ratio between the protein of interest and the total protein on the same blot. The use of stain-free imaging allows for the normalization of bands to the total protein on a blot, without requesting the use of housekeeping proteins or stripping and reprobing.

### 2.6. Statistical Analysis

All data were analyzed using the GraphPad Prism Program version 5.02. When the distribution of samples in each group respected the normal distribution, we used the parametric *t*-tests to compare two independent groups. When the distribution of samples did not respect a Gaussian distribution or was too small (*n* < 15), we used a nonparametric Mann–Whitney test to compare two independent groups. When more than two groups were compared, the nonparametric Kruskal–Wallis test was applied, followed by multiple comparison with Dunn's correction. In all cases, a *p* value < 0.05 was considered statistically significant.

## 3. Results

### 3.1. Clinical Features

One hundred and six patients were included in the study: 56 KC (25 corneal epithelium and 31 tears) and 50 healthy subjects (25 corneal epithelium and 25 tears). In the KC group, males represented 77.1%, with a mean age of 26.8 ± 8.5 years. KC rubbed eyes in 69.9%, with corneal opacity in 7.2%. In Amsler-Krumeich classification, grade 1, grade 2, grade 3, and grade 4 represented 22.9%, 45.8%, 9.6%, and 12.1%, respectively. In addition, forme fruste keratoconus represented 9.6%. In the control group, males represented 70.6%, with a mean age of 30.6 ± 8.1 years. No age-related or sex-related statistical differences were detected between the groups (*p* = 0.2). Statistical analysis stratified by age, gender, and KC severity did not reveal significant results (data not shown). More details of clinical and topographic features in each group are presented in Table 2.

### 3.2. Transcript Expression Levels of RAGE Isoforms and Ligands in KC and Healthy Epithelium

**Figure 3:**
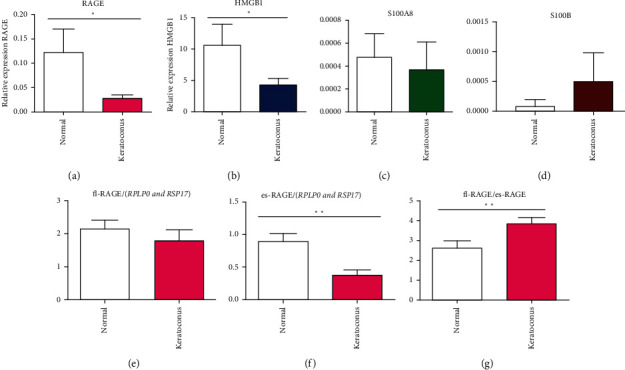
Transcript expression levels (mRNA) of total RAGE and its two principal isoforms (fl-RAGE and es-RAGE) and ligands in KC (25 corneal epithelium) and healthy subjects (25 corneal epithelium). RT-qPCR were performed to identify total RAGE isoforms (a) and his three major ligands (HMGB1 (b), S100A8 (c), and S100B (d)). In addition, we described the ratio between fl-RAGE (e) (transmembrane functional receptor responsible of inflammatory signaling) and es-RAGE (f) (soluble form of RAGE with an action of decoy, produced by alternative splicing) (g). Quantification of two housekeeping genes, RPLP0 and RPS17, and transcripts was performed for all samples as an internal control of the amount and quality of cDNA. The results are given as the ratio between the amount of each transcript of interest and the geometric mean of these two housekeeping genes (RPLP0 and RPS17) transcripts. ^∗^*p* < 0.05;  ^∗∗^*p* < 0.01.

The mRNAs were quantified in corneal samples for RAGE, HMGB1, and S100 family ligands and normalized with the geometric mean of two housekeeping genes' expressions (*RPLP0* and *RPS17*). Total RAGE showed a significant difference between normal and KC (Figure 3(a)). This was statistically higher in the normal group than in the KC group (*p* = 0.03). HMGB1, one of the most important ligands of RAGE, showed a similar expression profile (*p* = 0.04) (Figure 3(b)), whereas there were no significant differences for two S100 family ligands (Figures 3(c) and 3(d)). The ratio between fl-RAGE (functional transmembrane receptor responsible of inflammatory signaling) (Figure 3(e)) and es-RAGE (decoy of fl-RAGE involving anti-inflammatory response) (Figure 3(f)) was significantly higher in KC than in the control (*p* = 0.01) (Figure 3(g)). This difference was explained by a statistically lower es-RAGE expression in KC than control whereas there were no differences in fl-RAGE expression (*p* = 0.03).

### 3.3. Protein Expression Levels of RAGE Isoforms and Ligands in KC and Healthy Epithelium and Tears

**Figure 4:**
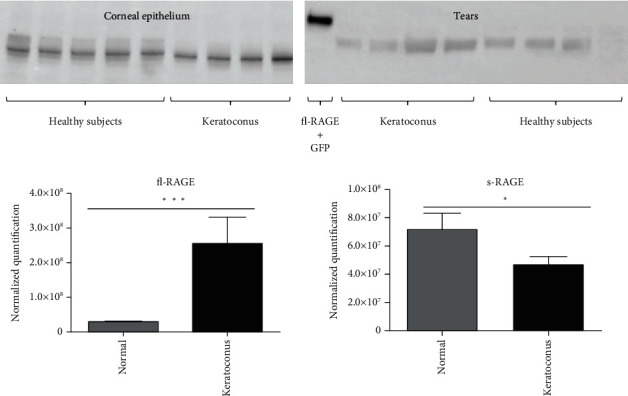
Western Blot of RAGE isoforms in keratoconus (25 corneal epithelium and 31 tears) and healthy subjects (25 corneal epithelium and 25 tears). Normalized quantification of fl-RAGE (transmembrane functional receptor of RAGE) in corneal epithelium samples (a) and s-RAGE (soluble forms of RAGE, with an action of decoy) in tears (b). Positive control chosen is transfected corneal cellular with fl-RAGE tagged GFP (expected molecular weight at 80kD). The relative intensities of protein bands were analyzed using Image Lab™ software (BIO-RAD), and the results were presented as a ratio between the protein of interest and the total protein on the same blot. ^∗^*p* < 0.05;  ^∗∗∗^*p* < 0.001.

fl-RAGE (transmembrane forms of RAGE) proteins were quantified in the corneal epithelium of KC and control. Quantification of the attempted band (molecular weight around 45-50 kD) showed a higher expression of fl-RAGE in the KC group than in the control group (*p* < 0.001) (Figure 4(a)). The s-RAGE (soluble forms of RAGE, decoy of fl-RAGE involving anti-inflammatory response) proteins were also quantified in tears of KC and compared with controls. Using the quantification of the attempted band (molecular weight around 30-35 kD) corresponding to s-RAGE, we established that the concentration of s-RAGE in tears was significantly higher in the normal group than in the KC group (*p* = 0.04). (Figure 4(b)).

## 4. Discussion

Keratoconus has been classically described as a noninflammatory corneal ectatic disorder. However, recent evidence suggests a possible role of inflammation in the pathogenesis of KC. Indeed, several articles have described an upregulation of proinflammatory cytokines, oxidative stress mediators, and enzymes in KC, without evidence in relation to biomarkers. The RAGE transmembrane receptor has been implicated in aseptic inflammation by reaction with AGEs, actors well-known in diabetes and cardiovascular disease. We describe here, for the first time in cornea and tears of KC, the implication of the RAGE-inflammatory pathway, with potential use of the related findings as potential biomarkers for this pathology.

### 4.1. Keratoconus: An Aseptic Inflammatory Disease?

KC was initially admitted to be a noninflammatory disease without cellular infiltration and vascularisation [2]. In the last few decades, many theories have been proposed for the pathogenesis of keratoconus such as incorrect development of cornea, genetic mutations, enzymes released by degeneration cells, and collagen disruption ([44–46]; Yaron S. [47]). Interestingly, extracellular matrix degradation is involved in the production of inflammatory cytokines and enzymes, and vice versa. Proinflammatory cytokines (IL-1, IL-6, TNF-*α*, and TGF-*β*) and enzymes, involved in degradation of collagens such as metalloproteases (MMP-1, MMP-3, and MMP-9), were described to be upregulated in KC when looking at tears and corneal samples [3, 21, 24, 25]. These proinflammatory cytokines seem to play a role in the protease cascade, along with the plasmin pathway, cyclooxygenase, and metalloproteinases, which may explain the observed changes in the extracellular matrix [48]. Considering that the production of MMPs is regulated by IL-1, IL-6, and IL-7 and that TNF-*α* increases MMP-1, MMP-3, and MMP-9, it was proposed that there is an inflammatory vicious circle established at the onset or during the progression of ectasia. In addition, evidence-based data showed that oxidative stress imbalance (in tears, cornea, aqueous humour, and blood) seemed to be a central process in the pathophysiology of KC [23]. Therefore, it appears that inflammation is clearly an integral part of the complex pathophysiology of KC.

### 4.2. RAGE: A Proinflammatory Pathway

RAGE is a glycoprotein transmembrane receptor binding AGEs or Damage-Associated Molecular Patterns (DAMPs also called alarmins): HMGB1/amphoterin family, S100/calgranulin family. RAGE is involved in various inflammatory processes like diabetic complications, chronic inflammatory diseases, atherosclerosis, and chronic neurodegenerative disease such as Alzheimer's disease [49]. A variety of tissues and cells express this pattern recognition receptor: vascular endothelial, bronchial, and pulmonary cells, vascular smooth muscle cells, neurons, and many others [29, 31]. The involvement of the RAGE pathway in corneal epithelial wound healing, as well as diseases of conjunctiva and retina and trabecular meshwork, has been demonstrated previously [35–41, 43]. The activation of fl-RAGE stimulates NF-*κ*B, a transcription factor residing at the inactive state in the cytosol of cells, which can modulate the expression of proinflammatory cytokines, vasoconstrictive and prothrombotic products, and adhesion molecules [29]. Truncated forms of RAGE are described as soluble decoys to prevent ligands from interacting with cell fl-RAGE surface receptor: es-RAGE (an alternative splicing product) and c-RAGE (a cleavage product by metalloproteases). Consequently, s-RAGE indirectly inhibits the fl-RAGE receptor. Thus, an inflammatory reaction mediated by fl-RAGE can be downregulated by these soluble decoys. This anti-inflammatory regulation has been described in bronchi and lungs of smokers, provoked by an underexpression of fl-RAGE with overexpression of es-RAGE isoforms [30, 50]. The inflammatory RAGE pathway is complex and still poorly understood because its activation can induce inflammatory cascades as well as anti-inflammatory retro control by soluble forms.

### 4.3. Dysregulation of RAGE Pathway in Keratoconus

**Figure 5:**
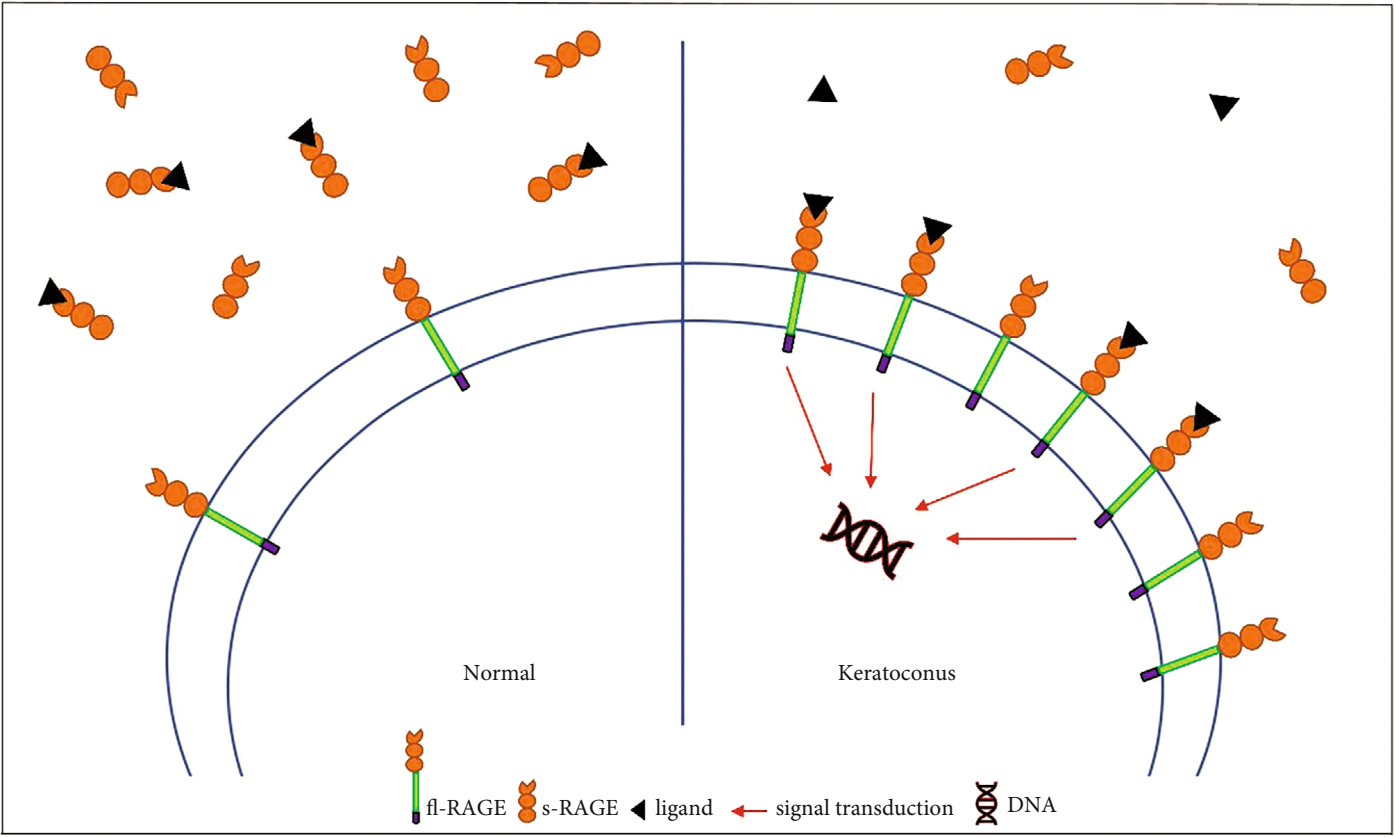
Feedback loop of inflammatory RAGE pathway: interaction between ligands, soluble decoys, and transmembrane receptors, in normal and KC cornea.

To our knowledge, our study described for the first time the implication of the RAGE pathway in KC. Results of RT-qPCR showed a significant underexpression of global RAGE transcripts in the corneal epithelium of keratoconus compared to healthy subjects. Nevertheless, this is emphasised by the ratio fl-RAGE/es-RAGE which highlighted a decrease of es-RAGE in KC (*p* = 0.01). Indeed, we did not observe any difference in fl-RAGE expression in the two groups using RT-qPCR whereas we highlighted a significant under-expression of es-RAGE in KC compared to controls. The results of Western Blotting showed a significant higher quantity of fl-RAGE protein in the corneal epithelium of KC compared to healthy subjects. These results were comforted by a significant lower quantity of s-RAGE protein (soluble forms) in tears of KC compared to healthy subjects. As explained above, the RAGE pathway can be implicated in activating inflammatory cascades as well as in an anti-inflammatory retro control, depending on tissues and pathologies [50]. In KC, the RAGE pathway seems to be upregulated in the corneal epithelium without upregulation of soluble decoys in tears. Interestingly, we highlighted a lower expression of RAGE transcripts concerning es-RAGE in KC, which could result from an alternative splicing mechanism. These results were comforted by RAGE-protein expression with a higher concentration of fl-RAGE in the corneal epithelium of KC and a lower concentration of s-RAGE (considered like decoys) in tears of KC compared with healthy patients. Our study shows that a proinflammatory dysregulation similar to that described for smokers in the lung is present in keratoconic corneas [30, 50]. In normal corneas, the activation of fl-RAGE (transmembrane receptor) can be downregulated by the soluble RAGE decoys while in KC there is an upregulation of the RAGE pathway without retro control of decoys in tears (Figure 5). Thus, the RAGE pathway is dysregulated in KC, potentially generating a number of proinflammatory signals in the ocular surface.

It is worth noting that the majority of KC in our study was grades 1 and 2 in Krumeich severity scale, which are the two lower stages of its ranking. It is possible that the RAGE pathway is present at an earlier stage of KC causing or fuelling the vicious circle of tissue remodelling. The modification of the expression of RAGE in early forms of keratoconus is particularly interesting: RAGE could be an easy access biomarker in tears particularly useful in keratoconus diagnosis. Indeed, KC is difficult to diagnose for nonspecialist ophthalmologists, requiring clinical and topographical evidences. In addition, the need for viable biomarkers in the early stages of KC is particularly interesting in preoperative of refractive surgery (where KC is contraindication) [51]. Further studies are needed to explore the inflammatory pathways resulting in the dysregulation of RAGE in KC and their putative implication in the progression of corneal ectasia. The biomarkers based on the RAGE pathway could become an interesting biological tool to evaluate KC progression. Moreover and interestingly, some blocking peptides like inhibitor ligands named RAP or SAGE and monoclonal specific antibodies anti-RAGE have been used in an attempt to block inflammatory RAGE pathway in different inflammation experimental models [32, 52–56]. The established biomarkers described in this study could also take a place for the future monitoring of RAGE-based therapeutics.

## 5. Conclusion

To our knowledge, we described for the first time the implication of RAGE pathway in KC. Other studies will compare these results and try to better specify the role that RAGE plays in KC pathophysiology and determine how the RAGE mRNA and protein isoforms (pro- or anti-inflammatory) could be used as future biomarkers for the diagnosis, prognosis, and treatment of the KC.

## Figures and Tables

**Table 1:** Forward and reverse primer sequences used for RT-PCR and RT-qPCR. These primer sequences were used in order to amplify human cDNAs of indicated genes obtained after reverse transcription of total mRNAs, extracted from the corneal epithelium of keratoconus and healthy subjects.

Gene	Sequence 5′-3′ (F: forward, R: reverse)	Product size (bp)	Hybridization temperature (°C)
Total RAGE	F: TGTGCTGATCCTCCCTGAGA	139	61
	R: CGAGGAGGGGCCAACTGCA		
es/fl-RAGE	F: TGTCAGCATCAGCATCATCG	fl-RAGE: 195 es-RAGE: 150	56
	R: TCCTGGTTTTCTGGGGCC		
hsRPLP0	F: AGGCTTTAGGTATCACCACT	219	61
	R: TATCACAGAGGAAACTCTGC		
hsRSP17	F: TGCGAGGAGATCGCCATTATC	169	61
	R: AAGGCTGAGACCTCAGGAAC		
hsHMGB1	F: ACCTATATCCCTCCCAAAGGG R: TTTTTGGGCGATACTCAGAGC	109	61
hsS100A8	F: TAAAGGGGAATTTCCATGCCGT R: GTTAACTGCACCATCAGTGTTG	137	61
hsS100B	F: AAGGGAGGGAGACAAGCACA R: TCCTGGAAGTCACATTCGCC	159	61

**Table 2:** Clinical features of keratoconus and healthy subjects.

			Clinical features						Topography			Amsler-Krumeich classification			
Samples	Population (*N*)	Age (years)	Sex (% men)	Eye rubbing (%)	Corneal opacity (%)	BCVA (logMar)	Km (D)	Kmax (D)	Astigmatism (D)	Thinnest P (*μ*m)	Suspect (%)	Grade 1 (%)	Grade 2 (%)	Grade 3 (%)	Grade 4 (%)
*Keratoconus*															
Corneal epithelium	25	24.3 (8.6)	78.8	69.7	3.0	0.23 (0.23)	45.8 (2.4)	56.9 (8.3)	1.33 (1.63)	476.2 (45.7)	0.0	30.3	57.6	6.1	6.7
Tears	31	28.5 (8.1)	72.1	70.2	10.0	0.20 (0.25)	48.6 (4.2)	51.3 (4.7)	6.50 (2.12)	453.5 (72.2)	16.0	18.0	38.0	12.0	16.0
Total	56	26.8 (8.5)	77.1	69.9	7.2	0.22 (0.24)	47.2 (3.5)	51.3 (4.7)	4.13 (2.17)	462.5 (63.7)	9.6	22.9	45.8	9.6	12.1
*Normal*															
Corneal epithelium	25	32.4 (6.4)	65.4	32.6	0.0	0.0	—	—	—	—	—	—	—	—	—
Tears	25	28.7 (9.8)	75.8	26.4	0.0	0.0	—	—	—	—	—	—	—	—	—
Total	50	30.6 (8.1)	70.6	29.6	0.0	0.0	—	—	—	—	—	—	—	—	—

Values are expressed as the mean (SD). BCVA: Best-Corrected Visual Acuity; D: Diopter.

## Data Availability

All the data used to support the findings of this study are included within the article and figures.

## Abbreviations

AGEs: Advanced glycation end products
c-DNA: Complementary-deoxyribonucleic acid
°C: Celsius degree
c-RAGE: Cleaved-RAGE
DAMPs: Damage-Associated Molecular Patterns
D: Diopter
dNTP: Deoxyribonucleotide
EDTA: Ethylenediaminetetraacetic acid
es-RAGE: Endogenous secretory-RAGE
fl-RAGE: Full length-RAGE
HMGB1: High-mobility group box 1
IL: Interleukin
KC: Keratoconus
Kmax: Maximal keratometry
ml: Milliliters
MMP: Matrix metalloproteinase
NaCl: Sodium Chloride
OCT: Optimal cutting temperature compound
PBS: Phosphate-Buffered Saline
PRK: Photorefractive keratectomy
RAGE: Receptor for Advanced Glycation End Products
RNA: Ribonucleic acid
RT-qPCR: Reverse Transcription Quantitative Polymerase Chain Reaction
SDS: Sodium dodecyl sulfate
s-RAGE: Soluble-RAGE
TBS: Tris-Buffered Saline
TBST: TBS and polysorbate 20 (Tween 20)
TGF: Tumoral growth factor
TNF: Tumoral necrosis factor
UVA: Ultraviolet A.
